# The safety and short-term effect of mixed approach in laparoscopic right hemicolectomy for right colon cancer compared with middle approach: a retrospective study

**DOI:** 10.1186/s12893-024-02405-3

**Published:** 2024-05-14

**Authors:** Shun-Yu Deng, Mao-Xing Liu, Pin Gao, Cheng-cai Zhang, Jia-Di Xing, Kechen Guo, Kai Xu, Fei Tan, Cheng-Hai Zhang, Ming Cui, Xiang-Qian Su

**Affiliations:** 1 Peking University Health Science Center, Beijing, 100038 China; 2 Key Laboratory of Carcinogenesis and Translational Research (Ministry of Education, Department of Gastrointestinal Surgery IV, Peking University Cancer Hospital & Institute, Haidian District, No.52 Fucheng Road, Beijing, 100142 China; 3 Zibo Center Hospital, Zibo, China

**Keywords:** Laparoscopic surgery, Colon cancer, Minimally invasive surgery, Surgery approaches

## Abstract

**Purpose:**

To investigate whether the mixed approach is a safe and advantageous way to operate laparoscopic right hemicolectomy.

**Methods:**

A retrospective study was performed on 316 patients who underwent laparoscopic right hemicolectomy in our center. They were assigned to the middle approach group (n = 158) and the mixed approach group (*n* = 158) according to the surgical approaches. The baseline data like gender、age and body mass index as well as the intraoperative and postoperative conditions including operation time, blood loss, postoperative hospital stay and complications were analyzed.

**Results:**

There were no significant differences in age, sex, BMI, ASA grade and tumor characteristics between the two groups. Compared with the middle approach group, the mixed approach group was significantly lower in terms of operation time (217.61 min vs 154.31 min, p < 0.001), intraoperative blood loss (73.8 ml vs 37.97 ml, p < 0.001) and postoperative drainage volume. There was no significant difference in the postoperative complications like postoperative anastomotic leakage, postoperative infection and postoperative intestinal obstruction.

**Conclusions:**

Compared with the middle approach, the mixed approach is a safe and advantageous way that can significantly shorten the operation time, reduce intraoperative bleeding and postoperative drainage volume, and does not prolong the length of hospital stay or increase the morbidity postoperative complications.

**Supplementary Information:**

The online version contains supplementary material available at 10.1186/s12893-024-02405-3.

## Background

Colorectal cancer is the third commonly diagnosed cancer and the second leading cause of cancer death worldwide, which represents a serious health hazard to mankind [1]. According to the latest cancer incidence statistics, the incidence of colorectal cancer is in the second place and the morality is the fourth among all malignant tumors in China, which is still on the rise in these years [2]. However, to date, surgery is the only way to radically cure the colorectal cancer.

Since the concept of complete mesocolic excision (CME) was proposed by R.J. Heald more than 20 years ago, it has been widely accepted and applied clinically. With the development of technology and minimally invasive concept, the therapeutic effect of laparoscopic surgery has also been proved clinically [3–5].

Although CME and D3 lymph node dissection have clarified the boundary of right hemicolectomy and the scope of lymph node dissection [6], there is still no standard approach to achieve this target. It is recognized that the middle approach is a safe and effective way to achieve CME and D3 lymph node dissection currently [7]. However, the operation takes a long time, and it is easy to cause more intraoperative bleeding [8]. In recent years, with the development of surgical technology, surgical approaches have been continuously optimized. There are many approaches applying in clinical, including cephalic approach, caudal approach and mixed approach, etc. [9–11]. However, in actual clinical practice, it may be difficult to accomplish one approach when encountering complex circumstance. Therefore, mixed approach is applied more widely. In order to further verify the safety and short-term effect of the mixed approach, we designed this study.

## Material

This study included patients who underwent laparoscopic right hemicolectomy from June 2010 to July 2021 at the IV Center of Gastrointestinal Surgery, Peking University Cancer Hospital. Inclusion criteria included: [1] patients over the age of 18 years (2) patients with pathological diagnosis of adenocarcinoma and preoperative clinical stage I, II, or III(3) patients were performed elective surgery rather than emergency surgery for perforation, severe obstruction or other emergency circumstances. While the exclusion criteria are: (1)the pathological diagnosis was not adenocarcinoma, including neuroendocrine carcinoma, spindle cell sarcoma, gastrointestinal stromal tumor, Hodgkin's lymphoma (2) multiple colorectal cancers; synchronous or metachronous cancers (3) receiving Endoscopic submucosal dissection (ESD), Endoscopic mucosal resection (EMR), neoadjuvant chemoradiotherapy or immunotherapy before this surgery (4) simultaneously resection of other organs(5) Open surgery.

**Fig. 1:**
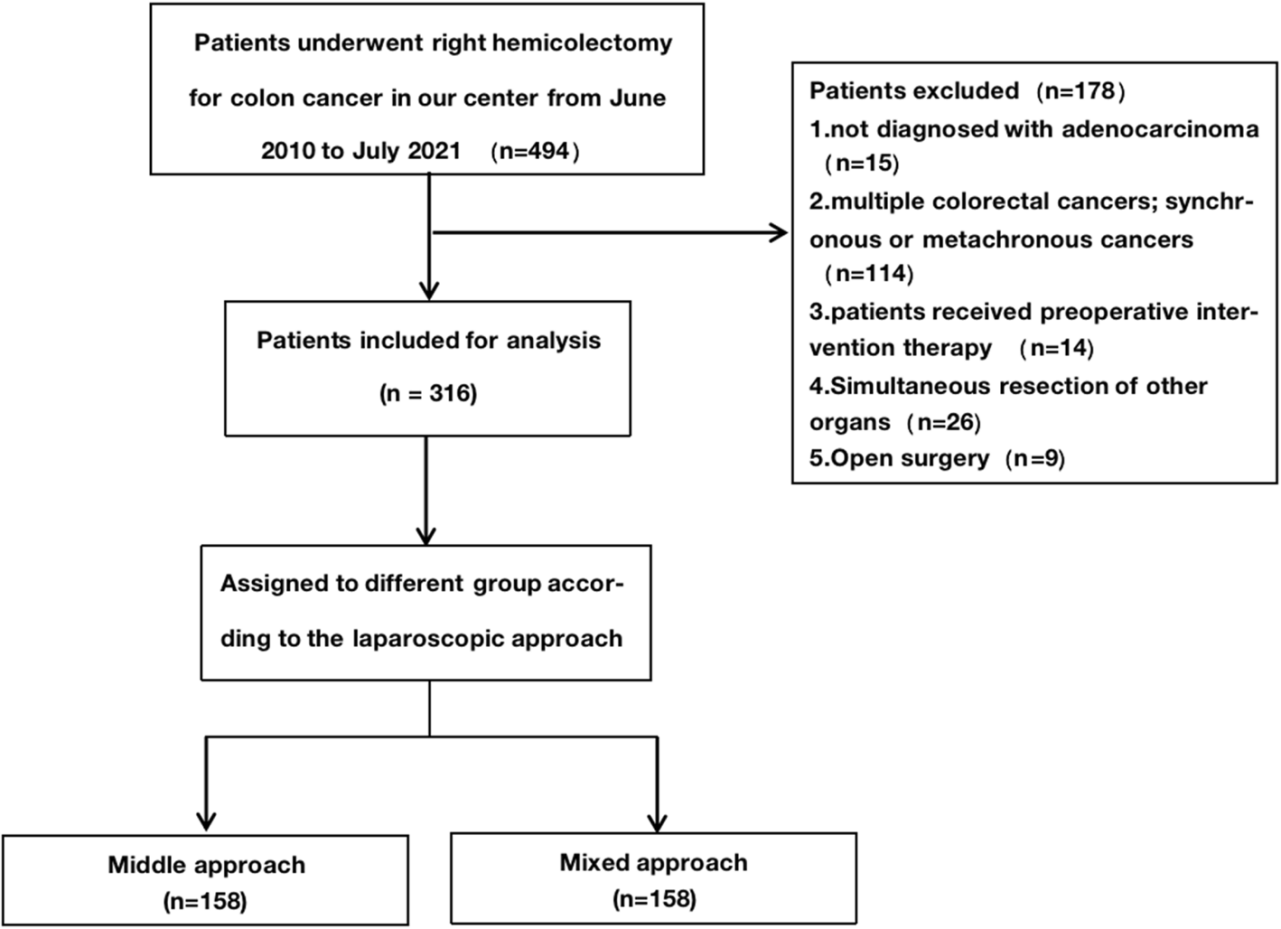
Flow chart of patients selection

A total of 494 patients underwent right hemicolectomy for colon cancer in our center from June 2010 to July 2021, and 15 of them were not pathologically diagnosed adenocarcinoma after surgery. 114 patients had at least two malignant tumors (including multiple colon malignant tumors); 14 patients received neoadjuvant therapy or ESD and other preoperative intervention therapy; Twenty-six patient’s surgery range ware more than right half of the colon; Open surgery for 9 patients. Finally, a total of 316 people were eventually enrolled in this experiment. (Fig. 1.)

Among them, 158 were performed with middle approach and 158 were performed with mixed approach. The operations were conducted by the team led by Professor Su Xiangqian. Both the mixed approach and the intermediate approach are commonly used by our team, and there is no obvious preference in their use. Choice between application of either the mixed approach or the middle approach was done by discretion of the consultant surgeon in charge preoperatively in each case. This choice has been sometimes corrected intraoperatively based on the surgeon's intraoperative considerations. The cases which initially used the middle approach but could not complete the central vessel dissection and ligation first due to technical difficulties and other reasons, changing the approach during the operation, were included in the mixed approach in this study. Because the main difference between the two approach is the order to process the central vessel. The baseline data of each group were listed in detail, including age, gender, body mass index (BMI), ASA grading, hypertension, diabetes, etc. In addition, we also analyzed the preoperative hemoglobin, oncology markers and other test results of patients. (Table 1) The patient's staging was based on the post-operative specimen biopsy and pathological staging according to AJCC standards. We also recorded tumor size, tumor site, degree of differentiation, vascular nerve invasion, and number of lymph nodes. (Table 2).

**Table 1:** Characteristics of the included patients

		Middle approach(*n* = 158)	Mixed approach(*n* = 158)	*P* value
Age(year)		59.98 ± 12.568	58.08 ± 12.754	0.182
Sex	Male	100	86	0.109
	Female	58	72	
BMI(Kg/m^2^)		23.98 ± 3.36	23.3 ± 3.14	0.067
Hypertention		51	45	0.463
Diabete		24	17	0.241
ASA	1	15	20	0.416
	2	134	125	
	3	9	13	
Abdominal surgery history		32	23	0.182
Preoperative intestinal obstruction		26	21	0.429
Preoperative hemoglobin concentration(g/L)		109.373 ± 22.65	109.734 ± 22.91	0.888
CEA(ng/ml)		15.08 ± 51.56	8.69 ± 14.58	0.173
CA199		35.33 ± 97.32	49.4 ± 159.89	0.368
CA724		15.34 ± 68.53	18.39 ± 80.46	0.74
CA242		20.13 ± 68.53	28.36 ± 64.34	0.228

**Table 2:** Pathological characteristics of the included patients

		Middle approach(*n* = 158)	Mixed approach(*n* = 158)	*P* value
Maximum tumor diameter(cm)		5.53 ± 2.40	5.48 ± 2.23	0.859
Minimum tumor diameter(cm)		4.09 ± 1.74	4.12 ± 1.74	0.889
Location	ileocecal	39	34	0.087
	ascending colon	67	86	
	hepatic flexure	52	38	
Differentiated degree	poor	10	21	0.144
	moderate	135	128	
	well	9	4	
Vessel invasive		37	27	0.262
Nerve invasive		43	28	0.059
Lymph node harvest		25.63 ± 11.29	25.98 ± 9.82	0.117
pTNM	1	21	14	0.09
	2	76	95	
	3	61	49	
T stge	1	9	3	0.133
	2	14	16	
	3	97	111	
	4	38	28	
N stage	0	97	109	0.283
	1	37	33	
	2	24	16	

### Surgical procedure

Laparoscopic right hemicoloectomy was completed in both groups of patients.In the end-to-side anastomosis group, an incision about 8 cm long was made in the middle of the upper abdomen, and the right half colon including tumor, mesocolon and sufficient intestinal segment were removed in vitro. End-to-side anastomosis of ileocolon (Johnson 28# stapler) was performed, and the stump was closed with a cutting closure device. The anastomosis was fixed and sutured, mesangium was closed and drainage tube was placed.

Mixed approach:The surgical methods were all conducted according to the preoperative routine intestinal preparation, accomplish related examinations, and strictly followed the diagnosis and treatment guidelines for colon cancer. After general anesthesia, the patient was placed in supine position with lower limbs separated. Pneumoperitoneum pressure was maintained at 12 ~ 14 mmHg. The five-hole method was performed and then explore the abdominal cavity and pelvic organs for metastatic nodules and ascites during the operation. After that, we examine the corresponding intestinal segment, find the primary site, identify and the tumor location, size, relationship with surrounding organs and mesenteric lymph nodes, mark the location of the lesion on the corresponding mesenteric tissues of the tumor, as well as determine the corresponding resection range. Localization methods include intraoperative fibro-colonoscopy and preoperative application of nano carbon labeling through colonoscopy, which can accurately locate the tumor. Open the right lateral peritoneum of the ascending colon to extend toldt’s space to the duodenum. Using ultrasonic knife, the mesocolon was incised along the vascular projection of the ileocolon. The arteries and veins of the ileocolon were also dissected. The surgical stem of the superior mesenteric vein was define, and the adipose tissue of the surrounding lymph nodes was dissected. The ileocolon artery, the right colon (with a low probability of occurrence) artery and the right branch of the middle colon artery were cut off on the right side of the surgical stem of the superior mesenteric vein from the caudal side to the cranial side. The posterior lobe of the right mesocolon was dissected along the right edge of the superior mesenteric vein. The anterior and posterior lobes of the mesocolon were completely resected along the fascia of Gerota and the anterior pancreatoduodenal fascia, and the lymphatic adiposed tissue in the mesangium was removed. The right omentum was excised by breaking the appetizing colic ligament. Then the hepatocolic ligament and the right phrenic colic ligament were cut off. Finally, the ascending colon and the hepatocolic region were dissociated.

Middle approach: First, along the anatomic projection of the ileocolic vessel pedicle. We anatomized the superior mesenteric vein and ligated the roots of the vessels. Then, following the fusion space of the hepatic flexure of the colon, the colonic hepatic flexure was completely dissected. Finally,the right colon was mobilized along with the expanded fusion fascia of Toldt.

### Observational indexes

Preoperative evaluation, including age, gende, BMI, American Society of Anesthesiologists Classification (ASA) [12] and abdominal surgery history etc., were recorded in detail. Intraoperative data included operation time, blood loss, specimen length, number of lymph nodes removed, and number of positive lymph nodes. Postoperative data were recorded, including defecation time, postoperative hospital stay and postoperative complications. Complications were graded according to Clavien-Dindo classification [13].

### Statistical analysis

All calculations and analyses were performed by SPSS software, version 22.0; Frequencies and percentages were used to describe the descriptive statistics with categorical variables. While quantitative variables were described as means and standard deviation (SD).The statistical significance of distribution differences in dichotomous variables was assessed using the Chi-squared test (χ2), whereas the Mann–Whitney test was used for ordinal values. P < 0.05 was considered statistically significant.

## Results

### Preoperative evaluation

A total of 316 patients with colon cancer were selected from 494 patients, including 158 in the mixed approach group and 158 in the middle approach group. The average age of the former was 58.08. While the latter was 59.98; There were no significant differences in hypertension, diabetes and functional evaluation (ASA grades) between the two groups. Considering the possibility that previous abdominal surgery or obstruction might affect the degree of intraoperative difficulty, this data was also collected to be analyzed. 51 patients with hypertension and 24 patients with diabetes in the middle approach, and 143 patients had ASA grades of grade II or above, there was no significant difference compared with the data in mixed approach group. Preoperative hemoglobin concentration and preoperative tumor markers are also listed in Table 1.

### Pathology characteristic

All postoperative specimens were pathologically examined and the pathologically related data was analyzed. In middle approach, the mean maximum tumor diameter was 5.53 cm, and the mean minimum tumor diameter was 4.09 cm. 39 cases were located in ileocecal part. 67 cases were located in ascending colon. 52 cases were located in hepatic curvature or transverse colon. In mixed approach, the mean length and diameter of tumors were 5.48 cm and 4.12 cm, respectively, and 34 cases were located in ileocecal part. 86 cases were located in ascending colon. 38 cases were located in hepatic curvature or transverse colon.

In terms of the degree of tumor differentiation, 10 patients in middle approach were in poor differentiation, and the rest were moderate or well, with 37 cases of vascular invasion and 43 cases of nerve invasion. In mixed approach, 21 cases were poor differentiation, including 27 cases of vascular invasion and 28 cases of nerve invasion.

According to the depth of tissue invasion and the number of lymph node metastases shown in the postoperative pathology of the patients, pathological staging was performed according to the AJCC tumor staging table. All patients included in our study were stage I, II or III, and there was no significant difference in the proportion of each stage between the two groups. The T stage and N stage were also listed in the table, as shown in the Table 2.

### Data related to surgery

The mean operation time in the middle approach group was 217.61 min, which was significantly higher than that in the mixed approach group (154.32 min, p < 0.001). The average amount of intraoperative blood loss in the mixed approach group was 37.97 ml, while that in the middle approach group was 73.8 ml, which was significantly higher than that in the mixed approach group (*p* < 0.001). In addition, 7 patients in the middle approach group received intraoperative blood transfusion treatment, while only 2 patients in the mixed approach group received blood transfusion treatment. The rate of blood transfusion blocked in middle approach group was higher than that in the mixed group, but there was no statistical significance (*p* = 0.091).

Rate of converting to open surgery: There was a possibility that laparoscopic surgery had to be transferred to open surgery due to intraoperative complex situations. Among them, 23 patients in the middle approach group conberted to open surgery, while only 13 patients in the mixed approach group converted to open surgery. Although the proportion of patients in the mixed approach group was relatively low, there was no significant statistical difference between the two groups.

For lymph node dissection,the mean number of all patients was 25.63 nodes in middle approach and 25.98 nodes in the mixed approach. The guidelines recommended that at least 12 lymph nodes be dissected. There were 7 patients with less than 12 lymph nodes dissected, 4 of whom were treated with a middle approach and 3 with a mixed approach. There was no statistically significant difference.

### Postoperative complications

There was no significant difference in the time with drainage tube between the two groups, which was 2.66 days in middle approach and 2.42 days in mixed approach. However, there was a statistically significant difference in the drainage volume between the two groups within 3 days after surgery. On the surgery day, the average drainage volume of the middle approach was 157.62 ml, while that of the mixed approach was 117.82 ml. (*p* = 0.001); On the first day after surgery, the average drainage volume of the middle approach was 218.05 ml, while that of the mixed approach was 178.11 ml. (*p* = 0.011); On the second day after surgery, the average drainage volume of the middle approach was 193.88 ml, while that of the mixed approach was 155.4 ml. (*p* = 0.016); On the third day after surgery, the average drainage volume of the middle approach was 198.33 ml, while that of the mixed approach was 149.62 ml. (*p* = 0.047);

In terms of gastrointestinal function, we recorded the time of patients' first defecation after surgery. The first defecation occurred at 4.22 days after surgery tin middle approach and 4.15 days in mixed approach (*p* = 0.085).

The rate of occurring anastomotic leakage, postoperative infection and intestinal obstruction were both relatively low in two groups. There was no significant statistical difference, and the patients with complications belonged to stage A and Stage B according to Clavein-Dindo stage which referred to no patients needed second surgery to treat complications after surgery(Table 3).

## Discussion

In recent years, the incidence of colorectal cancer has been increasing greatly. In colon cancer, the incidence of right colon cancer is higher. As the only radical treatment, the aim of surgery is to remove the intestinal segment where the tumor is located, central vascular ligation and lymphatic dissection. For patients who relapsed after surgery, the fundamental reason is that the operation did not completely achieve CMEs. Studies have pointed out that the prognosis of CME is very different from that of non-CME [14], so CME is a necessary condition for radical treatment. And how to better achieve CME has become a great concern of surgeons.

In middle approach, ileocolon vessels or superior mesenteric veins were used as indicators of laparoscopic anatomy. With the development of techniques, it’s reported that the 3D-reconstraction of mesenterial vascular anatomy helped to improvements of outcomes of the middle approach [15]. However, it is undeniable that this approach has practical difficulties in the exposure of mesenteric arteriovenous, identification of anatomical markers, and entry of anatomical space. In the process of expanding the right posterior colon space, the mesentery plain may be damaged due to the wrong level, and the retroperitoneal organs may also be injury. So the surgeons are required abundant experience. The mixed approach extends toldt’s space from the right side peritoneum of the ascending colon to the duodenum, which is more clear than middle approach and cause less intraoperative bleeding, thus reducing the difficulty of surgery. With the opening of the plane, blood vessel exposure is relatively easy. Compared with the middle approach, it can make up for the lack of clear identification of anatomical level by the middle approach and give full play to the advantages of vascular treatment, avoid the damage of adjacent tissues, so as to shorten the operation time, reduce intraoperative blood loss, and promote postoperative recovery.

Since the "No touch" principle was proposed in 1950, it has been widely used in the clinic to reduce the risk of recurrence and improve patient outcomes [14]. The technique prioritizes the ligation of central blood vessels, which reduces the risk of cancer cells spreading to the liver. In practice, iatrogenic spread of tumors includes intraoperative contact, which leads to direct spread to the peritoneum or other organs, resulting in postoperative recurrence. It can also cause postoperative liver metastasis by squeezing tumor cells directly through blood vessels into the portal system. This is also one of the reasons why the middle approach is recommended. However, an randomized controlled study (RCT) by Yasumasa Takii et al. (JCOG1006) pointed out that priority treatment of blood vessels may not significantly increase the prognosis [16], which further supported the rationality of the application of the mixed approach.

In the case of lymph dissection, studies have shown that the number of lymph nodes detected is associated with prognosis. Although there is still debate about the minimum number of lymph nodes to be removed, current guidelines recommend the detection of at least 12 or more lymph nodes [17]. There is no doubt that an increase in the number of lymph nodes detected contributes to a comprehensive assessment of lymph node involvement. In this way, the number of lymph node metastases can be more clearly determined, and the N stage can be more accurately determined, so as to give advice whether patients need postoperative adjuvant therapy according to the stage. For example, postoperative adjuvant therapy is recommended for stage III patients [17]. However, if the number of lymph nodes obtained is very small, the stage of patients may be inaccurate, and the postoperative adjuvant therapy may be missed, which means that the prognosis cannot be better. In addition, there has been controversy over the relationship between the number of lymph nodes detected and prognosis. Recent studies have pointed out that the increase in the number of lymph nodes obtained does not improve the detection rate of positive lymph nodes. Aisling et al.'s study pointed out that the lymph node production in colorectal cancer resection specimens increased [18]. However, this is not necessarily linked to an increase in the number of node-positive cancers. Currently, it is considered that factors related to the number of lymph nodes detected during colorectal cancer surgery, including tumor site size, length of surgical specimens, T-type classification, depth of invasion, and AJCC/UICC staging. They are predictive factors of the number of lymph nodes in colorectal cancer surgery [8, 10, 19]. However, several studies have indicated that the surgical approach is not correlated with the number of lymph nodes detected.

This studies still has several limitations. First, this study is a retrospective study in a single center. To confirm this conclusion, more RCTs are needed in the future. Secondly, the quality of surgical specimens was not uniformly assessed in this study. Finally, we only focused on the short-term outcomes while the long-term survival analysis was absent.

## Conclusion

From the perspective of membrane anatomy theory, the mixed approach has clearer anatomical layers and operating field that can significantly reduce the operation time, postoperative bleeding and postoperative drainage volume. Compared with the middle approach, it’s a safe and advantageous choice for laparoscopic right hemicolectomy. More RCTs are still needed to verify in the future.

### Supplementary Information

**Additional File 1. **Video of middle approach.

** Additional File 2. **Video of mixed approach.

** Additional File 3. **Reference 15.

## Data Availability

All data generated or analyzed during this study are included in this article and its additional files.
