# Supplementary material for: The safety and short-term effect of mixed approach in laparoscopic right hemicolectomy for right colon cancer compared with middle approach: a retrospective study
Source: BMC Surg. 2024 May 14;24:150. doi: 10.1186/s12893-024-02405-3 (PMC11092007; doi:10.1186/s12893-024-02405-3)
Supplement: Supplementary file 3 — Additional File 3. Reference 15. [file 12893_2024_2405_MOESM3_ESM.pdf]

International Journal of Colorectal Disease (2019) 34:151–160
https://doi.org/10.1007/s00384-018-3177-5

ORIGINAL ARTICLE

Preoperative anatomical road mapping reduces variability of operating time, estimated blood loss, and lymph node yield in right colectomy with extended D3 mesenterectomy for cancer

Christer-Daniel Willard [1] · Erik Kjaestad [1] · Bojan V. Stimec [2] · Bjorn Edwin [3,4] · Dejan Ignjatovic [1,4] · RCC Study Group

Accepted: 18 October 2018 / Published online: 1 November 2018
© Springer-Verlag GmbH Germany, part of Springer Nature 2018

Abstract

Objective To assess the impact of individual patient anatomy on operating time, estimated blood loss (EBL), and lymph node yield in right colectomy with extended D3 mesenterectomy, where surgeons have access to a preoperative 3-D reconstruction of the vascular anatomy of patients before surgery.

Aim/summary background data Data on the impact of individual patient vascular anatomy when surgeons have an anatomical road map as a guide at surgery is still missing in the literature.

Method Consecutive patients enrolled in an ongoing trial were classified into 4 groups and 2 subgroups using a 3-D vascular anatomy reconstruction derived from the staging CT. Outcome measures are operating time, EBL, vascular events, and D3 volume lymph node yield. SPSS was used for statistical analysis.

Results One hundred seventy-six (77 men) patients included. Mean operating time was 200 ± 50 min. Type 4b required significantly longer operating time (mean, 219 ± 59) compared to type 3 (mean, 188 ± 43) ($p = 0.004$). Vascular events occurred most often in anatomy type 4b (20.0%) and 3 (19.2%). No difference in EBL and lymph node yield was found ($p = 0.102$ and $p = 0.803$, respectively).

Conclusion The use of a roadmap at surgery seems to even differences in operating time, EBL, and lymph node yield, independent of the complexity of the individual patient's central mesenteric vascular anatomy. The incidents of vascular events requiring hemostasis do not cause differences in EBL between the anatomy groups, suggesting that preoperative awareness of the anatomy is beneficial at surgery.

Keywords Right colectomy · Colon cancer · 3D vascular anatomy · Surgical roadmapping · Operating time · Estimated Blood Loss · Lymph node yield

Introduction

Factors influencing operating time, estimated blood loss (EBL), and lymph node yield in colon cancer surgery can be viewed as disease-related, surgeon-related, and patient-related factors [1]. While the impact of factors such as disease stage, surgeons' re-training, experience, and/or volume can be simple to assess, this is not the case when individual vascular anatomy is the concern. Perhaps, even more concerning is the fact that the contribution of individual anatomy to the complexity of surgery has been too often accepted as an axiom, a statement up to date not corroborated within the literature [1].

On the other hand, a trend to increase lymph node yield—complete mesocolic excision (CME) with central vascular ligation (CVL), and D3 extended mesenterectomy anterior and posterior to the superior mesenteric vessels [2, 3]—is leading colorectal surgeons into regions of the human anatomy not previously dared. One such area, namely, the root of the mesentery, presents with unpredictable variations and/or abnormalities of the vascular anatomy [4, 5]. Lately, this venture was made possible through the use of modern high-resolution

✉ Dejan Ignjatovic
dexexer01@hotmail.com

1 Department of Digestive Surgery, Akershus University Hospital, University of Oslo, Lorenskog, Norway

2 Anatomy Sector, Department of Cell Physiology and Metabolism, Faculty of Medicine, University of Geneva, Geneva, Switzerland

3 Interventional Centre, Gastrointestinal and Pediatric Surgery, Oslo University Hospital–Rikshospitalet, Oslo, Norway

4 Institute of Clinical Medicine, University of Oslo, Oslo, Norway

radiology that depicts the individual patient's vascular anatomy through segmentation and 3D reconstruction [1, 4, 5]. The use of such anatomical roadmaps has been shown to significantly shorten operating time and lower EBL when compared to absence thereof [6, 7]. Moreover, this roadmap has been shown to have high specificity, sensitivity, precision, and accuracy [5, 7–10] and has been used to re-classify the variations and/or abnormalities of the superior mesenteric vessels into 4 groups and 2 subgroups, according to the crossing pattern of their branches [4, 5].

The aim of this study was to investigate the impact of the surgeons' preoperative awareness of individual patient's vascular anatomy on operating time, EBL, and lymph node yield in conventional right colectomy with extended D3 lymphadenectomy.

Material and methods

The subjects analyzed in this study are patients included into the safe D3 right hemicolectomy for cancer through multidetector computed tomography (MDCT) angiography trial (regional Ethical Committee approval REK Sør-Øst No. 2010/3354) and registered at clinicaltrials.gov (NCT01351714). Patients were required to sign an informed consent form prior to inclusion. The trial includes mandatory preoperative 3D reconstruction of the vascular anatomy, standardized definition of the D3 volume, standardized surgical approach to the central (level of dissection III) lymph nodes, and histopathological analysis of the D3 volume. All these data points were separately addressed below.

a) MDCT protocol and 3D reconstruction

Three-dimensional reconstruction of the vascular anatomy was derived from the preoperative staging MDCT. Anonymized CT datasets underwent segmentation and 3DVR, using the FDA-approved Osirix MD v. 8.5.2 64-bit image processing application (Pixmeo, Bernex, Switzerland), Mimics medical image processing software ver. 19.0.0.347, and 3-matic medical software ver. 11.0.0.109, both Windows 7 ultimate edition × 64 2016 (both Materialize NV, Leuven, Belgium). A large array of editing tools was applied (e.g., polygon, pixel revalidation, multiple slice editing, and dynamic region growing), enabling detailed linear and curved measurements. The data were exported as a report of the anatomy including vessel calibers and distances between arterial origins and venous confluences, images, and video clips, serving as a guide for preoperative planning.

b) Definition of the D3-volume

The third level of dissection (D3) volume was defined as mesenteric fatty tissue located anterior and posterior to the superior mesenteric vessels, limited by 4 lines connecting anatomical landmarks (vessel origins/confluences). The cranial border lies 5 mm proximal and parallel to the line connecting the confluence of the of the gastrocolic trunk of Henle (GTH) to the superior mesenteric vein (SMV) and the origin of the middle colic artery (MCA). The medial border runs along the left side of the superior mesenteric artery (SMA) from the origin of the MCA to the origin of the ileocolic artery (ICA). The caudal border is 10 mm distal to the line connecting the origin of the ICA and the confluence of the ileocolic vein (ICV) to the SMV. The lateral border lies 10 mm parallel to the right-hand side of the SMV [11–13] between the venous confluences of the ICV and GTH. This definition was consistently used for postoperative division of the surgical specimen into the respective D3/D2 areas of lymphadenectomy (Fig. 1).

c) Classification of the vascular anatomy

The classification into 4 vascular groups with 2 subgroups (Fig. 1) was according to the crossing patterns of colic arteries and jejunal vein (JV) as previously published [5]:

Type 1: ICA crosses anterior to the SMV, JV crosses anterior to the SMA
Type 2: ICA crosses anterior to the SMV, JV crosses posterior to the SMA
Type 3: ICA crosses posterior to the SMV, JV crosses posterior to the SMA
Type 4a: ICA crosses posterior to the SMV, JV crosses anterior to the SMA concealing the ICA origin
Type 4b: ICA crosses posterior to the SMV, JV crosses anterior to not concealing the ICA origin.

The previously reconstructed 3D images of the vascular anatomy were analyzed by two independent investigators (CDW and JMN) and classified into anatomical groups. In case of disagreement, a third investigator was consulted and a consensus reached (DI). In cases where no JV was found within the D3 area, the patient was classified as type 2 or 3 depending on ICA's crossing pattern.

d) The operative procedure

Surgical access was medial to lateral through a midline laparotomy. A transverse incision of the visceral peritoneum 1 cm distal to the origin of the ICA was made. The adipose tissue of the root of the mesentery was divided and the sheath of the terminal ileal venous trunk (TIVT) opened. The vascular sheath is then divided transversely towards the left; the SMA palpated, its vascular sheath opened, and the artery isolated. Vessel loops are placed around both vessels; the dissection continues through the division of the vascular sheath of

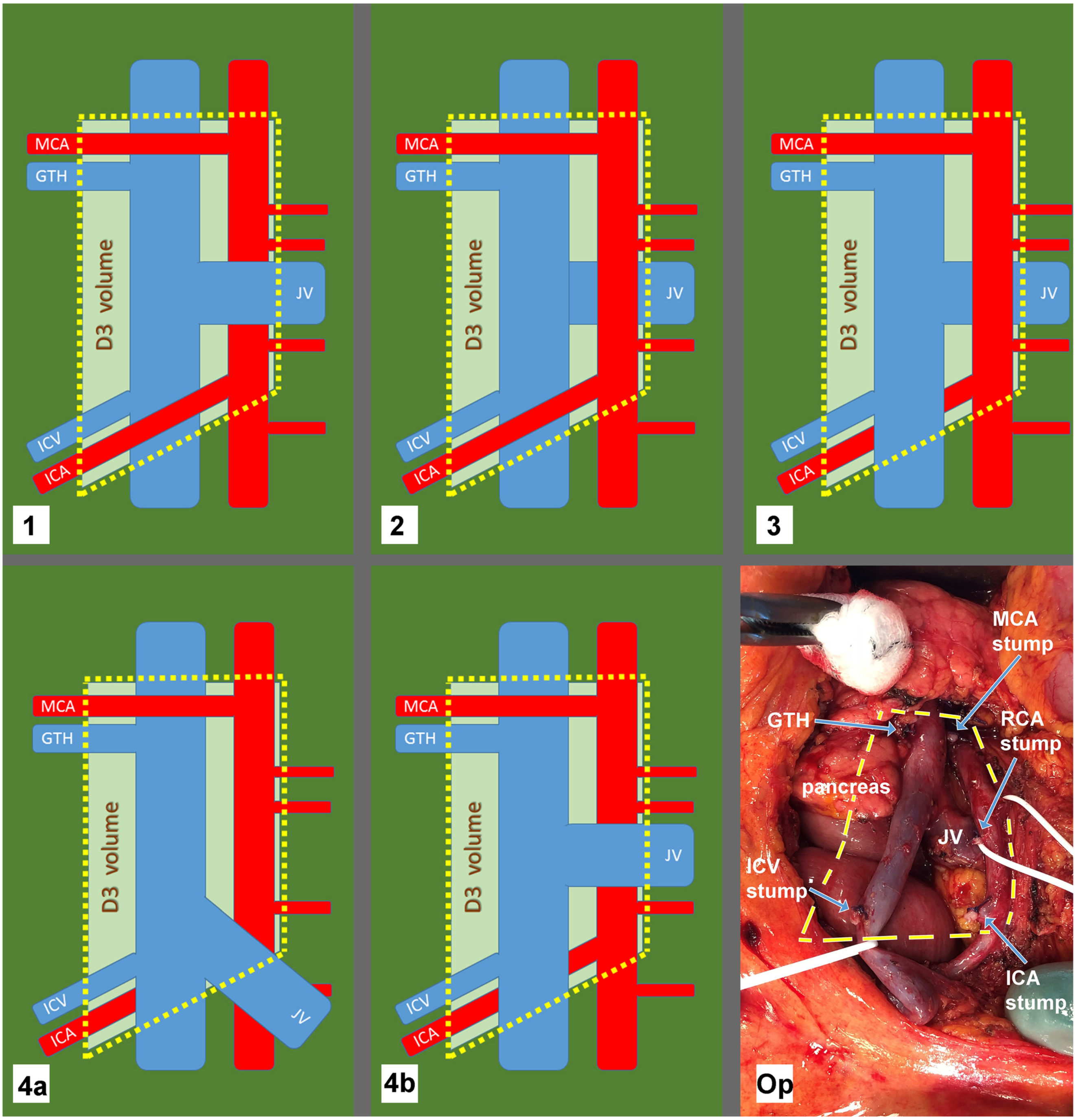

Fig 1 The vessels used as anatomical landmarks were the following: ICA, ileocolic artery; ICV, ileocolic vein; MCA, middle colic artery; GTH, gastrocolic trunk of Henle; JV, jejunal vein; SMV, superior mesenteric vein and SMA, superior mesenteric artery; and RCA, right colic artery. The interrupted yellow line depicts the D3 volume. (1) type 1, (2) type 2, (3) type 3, (4a) type 4a, (4b) type 4b, (Op) image at surgery. The SMV is lifted and drawn to the right, the SMA to the left showing that all tissue posterior to the superior mesenteric vessels has been removed.

the SMA along its left-hand side to the level of the MCA. The fatty tissue around the origin and along the trunk of the MCA was dissected towards the patient's right side, keeping the en-bloc specimen intact. The right branch of the MCA was divided. The anterior flap of the mesentery was lifted over the SMA and SMV and mobilized towards the right to the level of the GTH. The ICV and ICA were ligated. The SMV was lifted by gently pulling the vessel loop upwards, and its posterior aspect dissected free to the level of the MCA and GTH. This position was photographed from a right-angle view (with an etalon in place). Lateral mobilization of the de-vascularized specimen was then performed according to the principles of CME [2]. The mobilization included dissecting free the root of the mesentery from the horizontal duodenum, thus allowing access to

the posterior flap of mesenteric tissue. After the mobilization, the superior right colic vein was divided at its confluence with the GTH; the small bowel and colonic mesentery divided. The specimen (which was connected only through the posterior flap of mesenteric tissue) was dissected free and removed after the SMV was gently “rolled” towards the left and its vascular sheath together with the vascular sheath of the SMA divided with diathermy, removing complete arterial stumps.

e) The patients

Consecutive patients with resectable right colon cancer were prospectively evaluated from February 2011 to January 2017 in the above mentioned trial from two hospitals (Akershus University Hospital and Vestfold Hospital Trust). Data collection was prospective. Only patients meeting the following criteria were included in the analysis: (1) D3 right colectomy with extended mesenterectomy performed through laparotomy; (2) no resection of other organs; and (3) specimen divided into D3 and D2 areas of lymphadenectomy for histopathology purposes. The medical records were collected from the electronic medical record systems DIPS (Copyright 1995–2016 DIPS ASA version 7.395) and METAVISON (anesthesiology electronic record system included in DIPS). Three surgeons operated all patients (DI, YT, JMN).

f) Main outcome measures

Operating time was defined from time of incision to the last skin stitch. EBL was estimated based on the suction apparatus and weighing of surgical swabs. All surgical specimens underwent division by the senior surgeon into designated D2 and D3 area of lymphadenectomy according to the definition provided above, separately fixated in acetic acid, and sent to histopathological analysis stained with hematoxylin-eosin and safran.

Statistical analysis

Box plots were used to visualize differences between the anatomy groups. ANOVA was used to check for an interaction effect between the surgeon and the vascular anatomical groups. If there was a statistical significance using the F-test for an overall effect of vascular anatomy ($p < 0.05$), the LSD (least significant difference) post hoc test was used. Cohen kappa coefficient was used to assess the level of agreement between the researchers with regard to the vascular classification before a third observer was contacted. A general linear model with interaction effect (concerning vascular anatomy groups) was used to test the impact of BMI on operating time, EBL, and lymph node yield (LNY). All analyses were performed using the statistical software SPSS for Macintosh version 25.0 (SPSS Inc., Chicago, IL).

Results

The patients

From February 2011 to June 2017, 272 consecutive patients were enrolled in the abovementioned study. Of these, 176 (77 men) patients were eligible for the present study and underwent open right colectomy with extended D3 mesenterectomy. Ninety-six patients were excluded due to the following: right colectomy with D2 lymphadenectomy ($n = 12$), laparoscopic right colectomy with D2 lymphadenectomy ($n = 4$), laparoscopic right colectomy with D3 extended mesenterectomy ($n = 16$), surgical specimen not divided into the respective D3/D2 areas of lymphadenectomy ($n = 1$), R1/R2 resection ($n = 5$), and extended surgery ($n = 58$) including the following: (a) extended right colectomy (14), (b) extended small bowel resection (15), (c) subtotal colectomy (5), (d) multi-organ resection (16), (e) abdominal wall resection (3), and (f) transverse colon resection (2).

Mean patient age was 66 ± 9.6 years (mean ± standard deviation (SD)) and mean BMI was 25.9 ± 4.7 kg/m^2 (mean ± SD). Forty patients (22.5%) underwent previous surgery, 8 (4.5%) appendectomy, 11 (6.3%) hysterectomy, 6 (3.4%) cholecystectomy, cesarean section 1 (0.6%), and 14 (8.0%) other types of abdominal surgery.

Tumor location was in the cecum in 89 (50.8%), ascending colon in 73 (41.5%), hepatic flexure in 8 (4.5%), and proximal transverse colon in 4 (2.3%), and 2 (1.1%) patients had multiple tumors in the right colon.

Classification of patients in anatomy groups The level of agreement (Cohen’s kappa coefficient) between the independent reviewers (CDW and JMN) related to classifying the vascular types was 0.926 with a p value of $p < 0.001$. Patient numbers and demographic data for the anatomical groups and subgroups are shown in Table 1. The four major vascular groups were comparable for age, BMI, ASA scores (Fig. 2), gender ($p = 0.126$), and previous abdominal surgery ($p = 0.25$). BMI did not influence operating time (p = 0.45), EBL (p = 0.11), and D3 volume LNY (p = 0.18).

Operating time Mean operating time for all patients was 200 ± 50 (91–347) min. The interaction effect between surgeon and vascular anatomy group was not significant ($p = 0.588$), and the main effect for the vascular groups was not statistically significant ($p = 0.061$). When anatomy type 4 is split into the two subgroups (4a and 4b) and the five groups analyzed, no interaction effect between surgeon and vascular anatomy group ($p =$ 0.219) was found, but the main effect for the vascular groups was statistically significant ($p = 0.001$). The LSD post hoc test proved that patients with anatomy type 4b required statically longer operation times when compared to those in anatomy type 3: $p =$

Table 1 Patient numbers and demographic data for the anatomical groups and subgroups. Age: mean (standard deviation), median, and min–max. Sex: male/female. ASA score: American Society of Anesthesiologists Score. BMI with standard deviation and min-max

Anatomy group (*n*)	Age (mean (SD) (median) (min–max))	Sex (M/F)	ASA score	BMI (SD) (min–max)
Type 1 (11)	65 (11) (66) (36–76)	6/5	2 (1–3)	26.0 (3.3) (20.4–32.4)
Type 2 (51)	67 (10) (71) (39–89)	22/29	2 (1–4)	25.1 (3.8) (15.2–33.6)
Type 3 (52)	66 (8.3) (67,5) (45–81)	16/36	2 (1–4)	25.5 (5.6) (18.1–42.6)
Type 4 (62)	65 (10) (68) (34–86)	33/29	2 (1–3)	26.8 (4.7) (17.1–40.0)
Type 4a (37)	65 (10) (67) (44–78)	18/19	2 (1–3)	27.5 (4.9) (19.1–40.0)
Type 4b (25)	67 (10) (69) (44–86)	15/10	2 (1–3)	26.0 (4.4) (17.1–34.9)
Total (176)	66 (9.6) (68) (34–89)	77/99	2 (1–4)	26.0 (4.7) (15.2–42.6)

0.004 and 95% confidence interval (CI), 10–50 min. Operating times with *p* values and confidence intervals for all anatomy groups are listed in Tables 2 and 3 and Fig. 3.

EBL Mean EBL for all patients was 274 ± 304 (20–2530) mL. The interaction effect between surgeon and vascular anatomy group was not significant ($p = 0.567$), as well as the main effect for the vascular groups ($p = 0.945$). When dividing the type 4 group into 4a and 4b, there was no interaction effect between surgeon and vascular anatomy group ($p = 0.335$). The main effect for the vascular groups was not statistically significant ($p = 0.102$) (Table 2). There are two outliers in this analysis where major bleeding occurred: one in type 3 that was venous bleeding and one in type 4b that was an arterial bleed (Fig. 3).

Vascular events Twenty-two instances of bleeding requiring hemostasis were noted at surgery 0 in group 1, 6 in group 2 (11.8%), 10 in group 3 (19.2%), and 8 in group 4 (12.9%); 3 bleedings in group 4a (8.1%) and 5 in group 4b (20.0%) when divided into subgroups.

D3 volume lymph node yield The total number of lymph nodes for the whole surgical specimen was 40 ± 17 (6–101). The mean number of harvested lymph nodes in the D3 volume for the four vascular groups was 14 ± 10 (1–51) nodes, which represents 35% of lymph nodes harvested per specimen. The interaction effect between surgeon and vascular anatomy group was not significant ($p = 0.103$), and the main effect for the vascular groups was not statistically significant ($p = 0.853$). When dividing the type 4 into 4a and 4b, there was no interaction effect ($p = 0.75$) and the main effect was not statistically significant ($p = 0.803$) (see Table 2 and Fig. 3).

Discussion

The main finding of this study was that no differences in EBL or lymph node harvest were detected between the anatomical groups. The leveling of differences between the groups can be explained through the preoperative planning and the lack of the surprise factor at surgery. The results actually imply that the variability of operating time between the anatomical groups has been reduced with the exception of type 4b, which required significantly longer operating time (mean difference, 30 min). Data in the literature for operating times in open right colectomy are scarce showing values from 55 to 443 min [6, 15–18]. The longer operating times demonstrate the complexity of the D3 lymph node dissection.

The literature suggests significantly easier identification of the mesenteric vessels (5 vs. 23, $p < 0.001$) [6]; shorter operating times (17 min, $p = 0.027$) [6], (16 min, $p = 0.007$) [7]; lower frequency of intraoperative hemorrhage requiring hemostasis (6 vs. 19; $p = 0.006$) [6]; and no difference in lymph node harvest (4, $p = 0.067$) [7], (1, $p = 0.206$) [6] when performing a standard laparoscopic right colectomy (vascular ligation at the right-hand side of the SMV, D2 resection) for cancer with preoperative knowledge of the anatomy as compared to no preoperative awareness [6, 7, 19, 20].

When vascular events were analyzed separately, despite the fact that no statistical difference was found in mean EBL between the anatomical groups, a clear difference in number and percentage of vascular events requiring hemostasis was noted, the highest in anatomical group 4b (20.8%), separating this subgroup from the others. The fact that EBL was not higher in this group is most probably due to the preoperative awareness of the vascular anatomy that allows swift and appropriate action on the side of the operating surgeon, as compared to the surgeon that is facing a bleeding from an unknown vessel embedded in the mesenteric fat, and not having a 3-D reconstruction of the vascular anatomy. The longer operating time, the outliers, the reported vascular events, and the meticulous extended central mesenterectomy are contributing factors for a higher EBL. Data in the literature for EBL in open right colectomy with D3 lymph node dissection are scarce, showing values between 50 and 500 mL [16, 18, 21, 22].

Several elements at surgery can be the cause of prolonged operating time and/or increased EBL. The first and most essential step in the procedure is the correct positioning of the incision over the TIVT; an incision placed distally will require a larger dissection area and cause difficulty in orientation to the patients' anatomy, while a high incision will compromise the lymphadenectomy. When considering that the superior mesenteric vessels

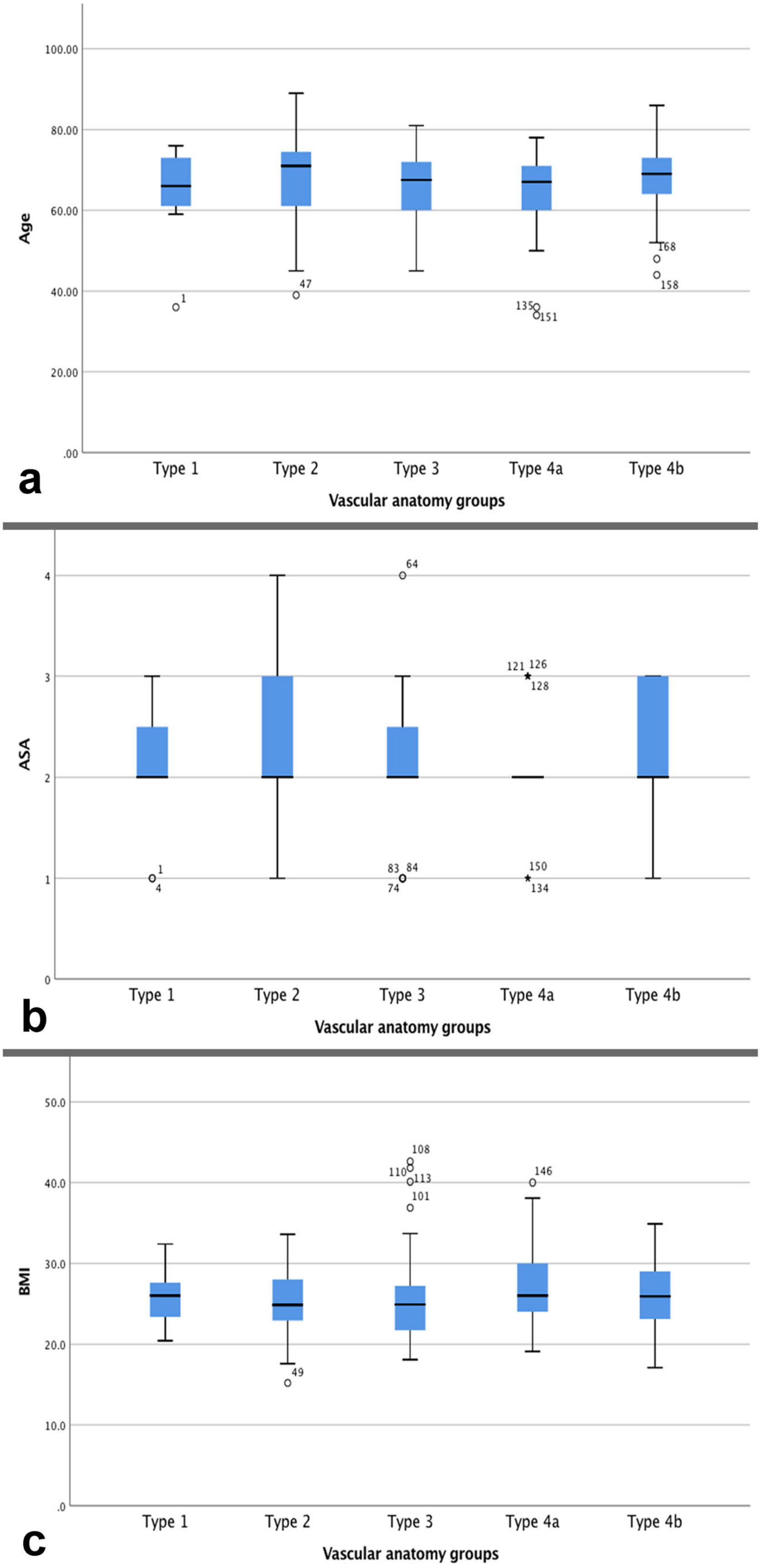

Fig. 2 Box-plots were used to demonstrate the comparability in age (**a**), ASA (**b**) and BMI (**c**) scores for patients in the anatomy groups

Table 2 All values are mean (standard deviation), min–max for operating times, estimated blood loss, and lymph node yield for anatomy groups

Anatomical group (*n*)	Type 1 (11)	Type 2 (51)	Type 3 (52)	Type 4 (61)	Type 4a (37)	Type 4b (25)	Total (176)
Operating time (min)	210 (41), 142–280	199 (47), 108–321	188 (43), 109–295	209 (59), 91–347	202 (58), 91–347	219 (59), 134–341	200 (50), 91–347
Blood loss (mL)	220 (185), 50–711	268 (251), 20–1300	311 (378), 50–2530	256 (294), 100–2000	190 (140), 100–670	354 (417), 100–2000	274 (304), 20–2530
Lymph node yield	15 (14), 1–48	13 (9), 1–51	14 (9), 1–42	15 (10), 3–47	15 (10), 3–47	14 (8), 2–34	14 (10), 1–51

are embedded in mesenteric fat and thus not readily visible in most patients, the roadmap provides orientation to the surgeon and the starting point for the surgery [5]. Moreover, the additional analysis using the general linear model with interaction effect failed to show correlation between BMI and operation time, EBL, and LNY, which can additionally be attributed to the use of the roadmap at surgery. The reason why patients with type 4b anatomy require most time is twofold: (1) a jejunal vein crossing the SMA anteriorly requires meticulous vascular dissection for its placement in a vessel loop (in order to allow progress in the dissection through the vascular sheath of the SMA towards the origin of the MCA); and (2) the position of this vein, a low lying vein can cause difficulty while accessing the origin of the ICA, while a higher crossing hampers safe access to the origin of the MCA. This dissection, in turn, carries a higher risk of bleeding, as our results clearly indicate. However, as far as EBL is concerned, it appears that neither the JV nor the occasional RCA in patients with type 4a or b anatomy cause difficulty when operating along the SMA and accessing the origin of the MCA. This can, again, be attributed to the 3D roadmap.

When lymph node yield was the concern, data derived from postmortem studies strongly suggests that lymph nodes can be found within the tissue anterior and posterior to the superior mesenteric vessels [12]. Moreover, these lymph nodes follow the crossing pattern of colic arteries. This implies that in a patient with an ICA crossing the SMV posteriorly, the nodes draining the right colon are also located posteriorly. The number of lymph nodes within this designated level of dissection III volume has

Table 3 Post hoc test for operating time with *p* values and confidence intervals for all anatomy groups.

Anatomy group		Mean difference	Std. error	Sig.	95% confidence interval	
					Lower bound	Upper bound
Type 1	Type 2	10.99	14.113	.437	− 16.88	38.86
	Type 3	21.29	14.089	.133	− 6.53	49.11
	Type 4a	7.85	14.579	.591	− 20.94	36.64
	Type 4b	− 9.12	15.360	.553	− 39.46	21.21
Type 2	Type 1	− 10.99	14.113	.437	− 38.86	16.88
	Type 3	10.30	8.367	.220	− 6.22	26.82
	Type 4a	− 3.14	9.168	.733	− 21.24	14.97
	Type 4b	− 20.11	10.365	.054	− 40.58	.36
Type 3	Type 1	− 21.29	14.089	.133	− 49.11	6.53
	Type 2	− 10.30	8.367	.220	− 26.82	6.22
	Type 4a	− 13.44	9.131	.143	− 31.47	4.59
	Type 4b	− 30.41*	10.332	.004	− 50.82	− 10.01
Type 4a	Type 1	− 7.85	14.579	.591	− 36.64	20.94
	Type 2	3.14	9.168	.733	− 14.97	21.24
	Type 3	13.44	9.131	.143	− 4.59	31.47
	Type 4b	− 16.98	10.991	.124	− 38.68	4.73
Type 4b	Type 1	9.12	15.360	.553	− 21.21	39.46
	Type 2	20.11	10.365	.054	− .36	40.58
	Type 3	30.41*	10.332	.004	10.01	50.82
	Type 4a	16.98	10.991	.124	− 4.73	38.68

*Type 3 and type 4b statistical significant difference

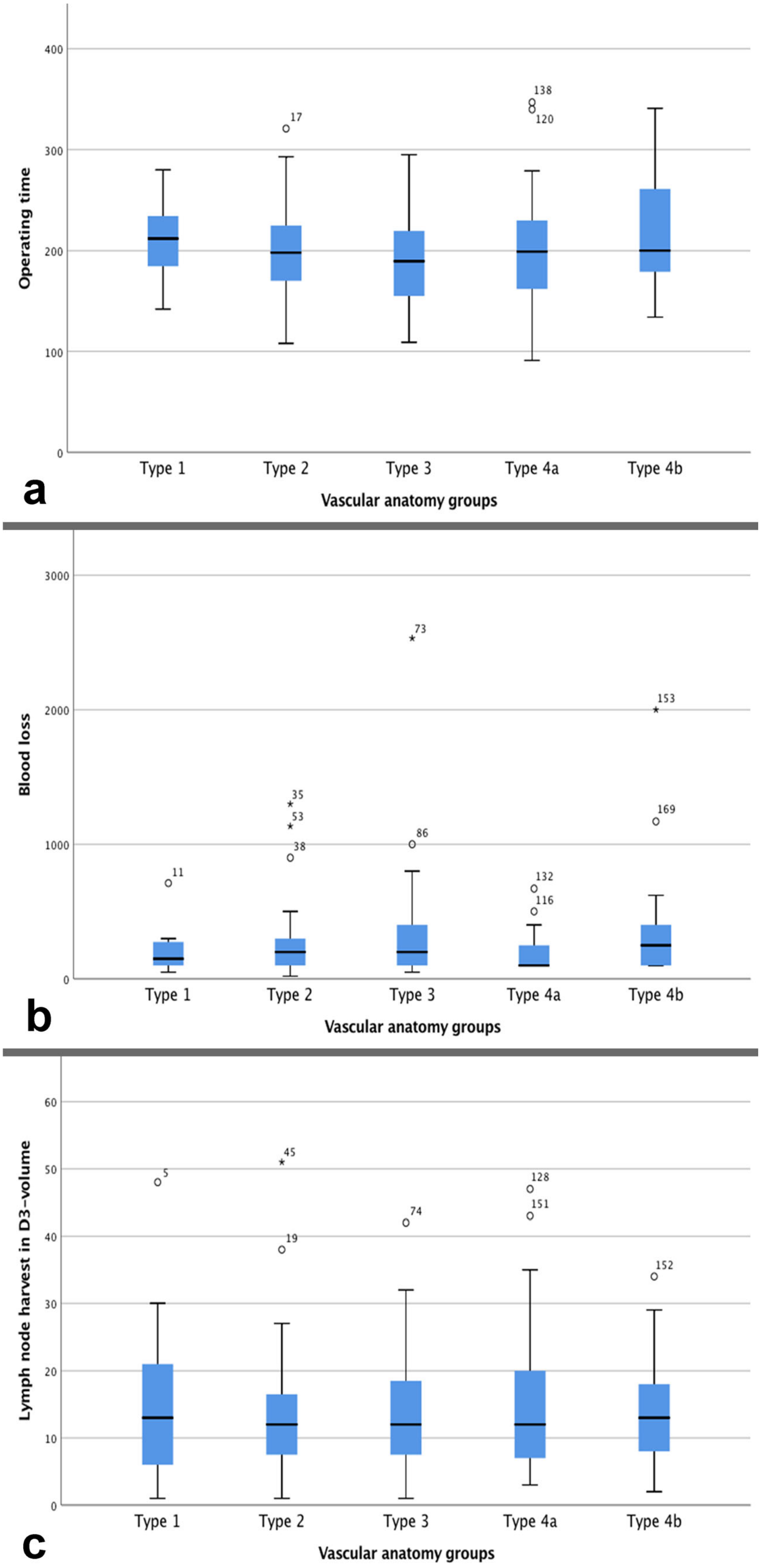

Fig 3 Box and whiskers plot for operating time (**a**), EBL (**b**), and lymph node yield (**c**) between the anatomy groups

been reported to be 15.9 ± 7.4 [11]. A search in the literature did not provide any data on isolated D3 volume lymph node yield, but only on the whole specimen showing values between 2 and 169 [2, 3, 6, 14–16, 18, 21–28]. Our results imply that the result of such a complete lymphadenectomy for right sided colon cancer includes 14 ± 10 lymph nodes within the D3 volume and that these do not depend on the individual patient anatomy, but rather on the operative procedure performed (the extent of mesenterectomy), in this manner, securing a steady lymph node harvest in all patients [11, 12]. Possibly even more important is the approach to the D3 volume from outside its borders, allowing a true complete mesocolic excision and central vascular ligation. In this manner, no spillage of the colonic lymph (potentially containing isolated cancer cells, sub-micro or micro-metastases) occurs through dissection to the left of the watershed of the small bowel lymph [12]. The main interest of this article is the dissection of the D3 volume lymph nodes which is performed first to devascularize the surgical specimen, while the right colectomy was always performed after devascularization as a standardized lateral to medial mobilization of the surgical specimen respecting the embryological plane, according to the principles of CME as originally described by Hohenberger et al. [2].

Drawbacks of this study lie in the fact that mobilization of the coherent surgical specimen is necessary in order to remove also the tissue posterior to the superior mesenteric vessels, namely, the use of time from incision to the last stitch and not only a partial operating time. This decreases the chances for detection of significant differences between the anatomy groups in operating time, while simultaneously confirming that only the significant difference was actually found. Another limitation of this study is the low number of patients in anatomical group 1 and that a control group with surgeons unaware of the anatomy before surgery is missing. Due to the possible pitfalls while preforming surgical procedures in the central mesentery, namely, removing all fatty tissue both in front and behind the SMA and SMV, preoperative awareness of the branching patterns of these vessels is deemed necessary in order to achieve safe hemostasis when the need occurs. This is why the regional ethical committee approval states that preoperative awareness of the individual patient's anatomy is mandatory. Achieving hemostasis while not being aware of the anatomy is hazardous and is the reason that a control group for this study is unethical. This is confirmed through an incidence of 20.8% vascular events requiring hemostasis in patients having anatomy type 4b as shown through our results.

It is, therefore, of great importance when performing right colectomy with extended D3 mesenterectomy anterior and posterior to the superior mesenteric vessels to apprehend the individual venous and arterial variants for safe vascular dissection [6, 7, 9, 19, 22].

The strength of the study lies in the setting of a prospective design, uniform operative technique, and that preoperative 3D anatomical reconstruction was preformed by only one author (BVS).

Conclusion

The use of a *roadmap* at surgery seems to even differences in operating time, EBL, and lymph node yield, independent of the complexity of the individual patient's central mesenteric vascular anatomy. The incidents of vascular events requiring hemostasis do not cause differences in EBL between the anatomy groups, suggesting that preoperative awareness of the anatomy is beneficial at surgery.

Acknowledgements The Right Colon Cancer Study Group (RCC-group): Prof. Dr. Tom Oresland, MD, Akershus University Hospital, Department of Digestive Surgery, University in Oslo, Norway; Prof. Dr. Arne O. Bakka, MD, Akershus University Hospital, Department of Digestive Surgery, University in Oslo, Norway; Assoc Prof. Ola Reiertsen, MD, Akershus University Hospital, Department of Digestive Surgery, University in Oslo, Norway; Arne Engebreth Færden, MD, PhD, Akershus University Hospital, Department of Digestive Surgery, Oslo, Norway; Yngve Thorsen, MD, Akershus University Hospital, Department of Digestive Surgery, University in Oslo, Norway; Prof. Solveig Norheim Andersen, MD, Akershus University Hospital, Department of Pathology, University in Oslo, Norway; Anne Negaard, MD, PhD, Akershus University Hospital, Department of Radiology, Oslo, Norway; Aly Dicko, MD, Department of Digestive Surgery, Haukeland University Hospital, Bergen, Norway; Prof. Frank Pfeffer, MD, Department of Digestive Surgery, Haukeland University Hospital, Bergen, Norway; Hovard Forsmo, MD, Department of Digestive Surgery, Haukeland University Hospital, Bergen, Norway; Sigmund Ytre-Hauge, MD, Department of Radiology, Haukeland University Hospital, Bergen, Norway; Jens Marius Nesgaard MD, Department of Digestive Surgery, Vestfold Hospital Trust, Tonsberg, Norway; Russel Jacobsen, MD, Department of Vascular Surgery, Vestfold Hospital Trust, Tonsberg, Norway; Kari Mette Langerød von Brandis, MD, Department of Radiology, Vestfold Hospital Trust, Tonsberg, Norway; Tania Hansen, Department of Radiology, Vestfold Hospital Trust, Tonsberg, Norway; Pål Suhrke, MD, Department of Pathology, Vestfold Hospital Trust, Tonsberg, Norway; Javier Luzon, MD, Akershus University Hospital, Department of Digestive Surgery, University in Oslo, Norway; Barış Sevinç, MD, Department of General Surgery, Medical Park Uşak Hospital, Uşak, Turkey; Bjarte Tidemann Andersen, MD, Department of Digestive Surgery, Østfold Hospital Trust, Grålum, Norway; Robin Gaupset, MD, Department of Digestive Surgery, Akershus University Hospital, Oslo, Norway; Prof. Dr. Roberto Bergamaschi, MD, Division of Colorectal Surgery, Westchester Medical Center, New York Medical College, NY, USA; Frieder Pullig, MD Viszeralchirurgie Klinikum Karlsruhe Germany; Ulrich Schneider, MD Institut für Pathologie Klinikum Karlsruhe, Germany; Joerg Baral, MD Viszeralchirurgie Klinikum Karlsruhe Germany; Marcos Gomez Ruiz MD, Coloproctologia Cirugia Colorrectal - Cirugía General y Ap. Digestivo Hospital Universitario Marques de Valdecilla, Spain; Jonas Lindstrøm Health Services Research Unit, Akershus University Hospital, Lorenskog, Norway; Ariba Ehsan Sheikh, MD, Akershus University Hospital, Department of Digestive Surgery, University in Oslo, Norway; Tine Strommen, MD, Akershus University Hospital, Department of Digestive Surgery, University in Oslo, Norway.

Authors' contribution All authors contributed to the conception, design, and data collection. The authors have actively participated both in writing parts of the manuscript and in revising it, and in the end, have given final approval for the manuscript to be published. The study conducted is a cohort study.

Compliance with ethical standards

Conflict of interest The authors declare that they have no conflict of interest.

References

1. Miyamoto R, Tadano S, Sano N, Inagawa S, Adachi S, Yamamoto M (2017) The impact of three-dimensional reconstruction on laparoscopic-assisted surgery for right-sided colon cancer. Wideochir Inne Tech Maloinwazyjne 12(3):251–256
2. Hohenberger W, Weber K, Matzel K, Papadopoulos T, Merkel S (2009) Standardized surgery for colonic cancer: complete mesocolic excision and central ligation–technical notes and outcome. Colorectal Dis 11(4):354–364 discussion 64-5
3. West NP, Hohenberger W, Weber K, Perrakis A, Finan PJ, Quirke P (2010) Complete mesocolic excision with central vascular ligation produces an oncologically superior specimen compared with standard surgery for carcinoma of the colon. J Clin Oncol Off J Am Soc Clin Oncol 28(2):272–278
4. Nesgaard JM, Stimec BV, Bakka AO, Edwin B, Ignjatovic D (2017) Navigating the mesentery: part II. Vascular abnormalities and a review of the literature. Colorectal Dis 19(7):656–666
5. Nesgaard JM, Stimec BV, Bakka AO, Edwin B, Ignjatovic D (2015) Navigating the mesentery: a comparative pre- and peroperative visualization of the vascular anatomy. Colorectal Dis 17(9):810–818
6. Mari FS, Nigri G, Pancaldi A, De Cecco CN, Gasparrini M, Dall'Oglio A et al (2013) Role of CT angiography with three-dimensional reconstruction of mesenteric vessels in laparoscopic colorectal resections: a randomized controlled trial. Surg Endosc 27(6):2058–2067
7. Wang JB, Huang CM, Zheng CH, Li P, Xie JW, Lin JX, Lu J (2014) Role of 3DCT in laparoscopic total gastrectomy with spleen-preserving splenic lymph node dissection. World J Gastroenterol 20(16):4797–4805
8. Takiguchi S, Sekimoto M, Fujiwara Y, Yasuda T, Yano M, Hori M, Murakami T, Nakamura H, Monden M (2004) Laparoscopic lymph node dissection for gastric cancer with intraoperative navigation using three-dimensional angio computed tomography images reconstructed as laparoscopic view. Surg Endosc 18(1):106–110
9. Kanamoto T, Matsuki M, Okuda J, Inada Y, Tatsugami F, Tanikake M, Yoshikawa S, Narabayashi I, Kawasaki H, Tanaka K, Yamamoto T, Tanigawa N, Egashira Y, Shibayama Y (2007) Preoperative evaluation of local invasion and metastatic lymph nodes of colorectal cancer and mesenteric vascular variations using multidetector-row computed tomography before laparoscopic surgery. J Comput Assist Tomogr 31(6):831–839
10. Sakaguchi T, Suzuki S, Morita Y, Oishi K, Suzuki A, Fukumoto K, Inaba K, Kamiya K, Ota M, Setoguchi T, Takehara Y, Nasu H, Nakamura S, Konno H (2010) Analysis of anatomic variants of mesenteric veins by 3-dimensional portography using multidetector-row computed tomography. Am J Surg 200(1):15–22
11. Spasojevic M, Stimec BV, Dyrbekk AP, Tepavcevic Z, Edwin B, Bakka A et al (2013) Lymph node distribution in the d3 area of the right mesocolon: implications for an anatomically correct cancer resection. A postmortem study. Dis Colon Rectum 56(12):1381–1387
12. Nesgaard JM, Stimec BV, Soulie P, Edwin B, Bakka A, Ignjatovic D (2018) Defining minimal clearances for adequate lymphatic resection relevant to right colectomy for cancer: a post-mortem study. Surg Endosc 32:3806–3812
13. Negoi I, Beuran M, Hostiuc S, Negoi RI, Inoue Y (2018) Surgical anatomy of the superior mesenteric vessels related to colon and pancreatic surgery: a systematic review and meta-analysis. Sci Rep 8(1):4184
14. Liang JT, Lai HS, Huang J, Sun CT (2015) Long-term oncologic results of laparoscopic D3 lymphadenectomy with complete mesocolic excision for right-sided colon cancer with clinically positive lymph nodes. Surg Endosc 29(8):2394–2401
15. Feng B, Sun J, Ling TL, Lu AG, Wang ML, Chen XY, Ma JJ, Li JW, Zang L, Han DP, Zheng MH (2012) Laparoscopic complete mesocolic excision (CME) with medial access for right-hemi colon cancer: feasibility and technical strategies. Surg Endosc 26(12): 3669–3675
16. Bae SU, Saklani AP, Lim DR, Kim DW, Hur H, Min BS, Baik SH, Lee KY, Kim NK (2014) Laparoscopic-assisted versus open complete mesocolic excision and central vascular ligation for right-sided colon cancer. Ann Surg Oncol 21(7):2288–2294
17. Pelz JOW, Wagner J, Lichthardt S, Baur J, Kastner C, Matthes N, Germer CT, Wiegering A (2018) Laparoscopic right-sided colon resection for colon cancer-has the control group so far been chosen correctly? World J Surg Oncol 16(1):117
18. Lee SD, Lim SB (2009) D3 lymphadenectomy using a medial to lateral approach for curable right-sided colon cancer. Int J Color Dis 24(3):295–300
19. Ignjatovic D, Sund S, Stimec B, Bergamaschi R (2007) Vascular relationships in right colectomy for cancer: clinical implications. Tech Coloproctol 11(3):247–250
20. Natsume T, Shuto K, Yanagawa N, Akai T, Kawahira H, Hayashi H, Matsubara H (2011) The classification of anatomic variations in the perigastric vessels by dual-phase CT to reduce intraoperative bleeding during laparoscopic gastrectomy. Surg Endosc 25(5): 1420–1424
21. Galizia G, Lieto E, De Vita F, Ferraraccio F, Zamboli A, Mabilia A et al (2014) Is complete mesocolic excision with central vascular ligation safe and effective in the surgical treatment of right-sided colon cancers? A prospective study. Int J Color Dis 29(1):89–97
22. Ogino T, Takemasa I, Horitsugi G, Furuyashiki M, Ohta K, Uemura M et al (2014) Preoperative evaluation of venous anatomy in laparoscopic complete mesocolic excision for right colon cancer. Ann Surg Oncol 21(Suppl 3):S429–S435
23. Storli KE, Sondenaa K, Furnes B, Nesvik I, Gudlaugsson E, Bukholm I et al (2014) Short term results of complete (D3) vs. standard (D2) mesenteric excision in colon cancer shows improved outcome of complete mesenteric excision in patients with TNM stages I-II. Tech Coloproctol 18(6):557–564
24. Kessler H, Hohenberger W (2013) Extended lymphadenectomy in colon cancer is crucial. World J Surg 37(8):1789–1798
25. Kotake K, Mizuguchi T, Moritani K, Wada O, Ozawa H, Oki I, Sugihara K (2014) Impact of D3 lymph node dissection on survival for patients with T3 and T4 colon cancer. Int J Color Dis 29(7):847–852
26. Munkedal DL, West NP, Iversen LH, Hagemann-Madsen R, Quirke P, Laurberg S (2014) Implementation of complete mesocolic excision at a university hospital in Denmark: an audit of consecutive, prospectively collected colon cancer specimens. Eur J Surg Oncol 40(11):1494–1501
27. Kotake K, Kobayashi H, Asano M, Ozawa H, Sugihara K (2015) Influence of extent of lymph node dissection on survival for patients with pT2 colon cancer. Int J Color Dis 30(6):813–820
28. Cho MS, Baek SJ, Hur H, Soh Min B, Baik SH, Kyu Kim N (2015) Modified complete mesocolic excision with central vascular ligation for the treatment of right-sided colon cancer: long-term outcomes and prognostic factors. Ann Surg 261(4):708–715
